# Predictors of medical school clerkship performance: a multispecialty longitudinal analysis of standardized examination scores and clinical assessments

**DOI:** 10.1186/s12909-016-0652-y

**Published:** 2016-04-27

**Authors:** Petra M. Casey, Brian A. Palmer, Geoffrey B. Thompson, Torrey A. Laack, Matthew R. Thomas, Martha F. Hartz, Jani R. Jensen, Benjamin J. Sandefur, Julie E. Hammack, Jerry W. Swanson, Robert D. Sheeler, Joseph P. Grande

**Affiliations:** Division of Gynecology, Mayo Clinic, 200 First St SW, Rochester, MN 55905 USA; Department of Psychiatry and Psychology, Mayo Clinic, 200 First St SW, Rochester, MN 55905 USA; Division of Subspecialty General Surgery, Mayo Clinic, 200 First St SW, Rochester, MN 55905 USA; Department of Emergency Medicine, Mayo Clinic, 200 First St SW, Rochester, MN 55905 USA; Department of Medicine, Mayo Clinic, 200 First St SW, Rochester, MN 55905 USA; Department of Pediatrics and Adolescent Medicine, Mayo Clinic, 200 First St SW, Rochester, MN 55905 USA; Division of Reproductive Endocrinology and Infertility, Mayo Clinic, 200 First St SW, Rochester, MN 55905 USA; Department of Neurology, Mayo Clinic, 200 First St SW, Rochester, MN 55905 USA; Department of Family Medicine, Mayo Clinic, 200 First St SW, Rochester, MN 55905 USA; Department of Anatomic Pathology and Laboratory Medicine, Mayo Clinic, 200 First St SW, Rochester, MN 55905 USA

**Keywords:** Assessment, Clerkship, Clinical rotation, Standardized scores

## Abstract

**Background:**

Evidence suggests that poor performance on standardized tests before and early in medical school is associated with poor performance on standardized tests later in medical school and beyond. This study aimed to explore relationships between standardized examination scores (before and during medical school) with test and clinical performance across all core clinical clerkships.

**Methods:**

We evaluated characteristics of 435 students at Mayo Medical School (MMS) who matriculated 2000–2009 and for whom undergraduate grade point average, medical college aptitude test (MCAT), medical school standardized tests (United States Medical Licensing Examination [USMLE] 1 and 2; National Board of Medical Examiners [NBME] subject examination), and faculty assessments were available. We assessed the correlation between scores and assessments and determined USMLE 1 cutoffs predictive of poor performance (≤10th percentile) on the NBME examinations. We also compared the mean faculty assessment scores of MMS students vs visiting students, and for the NBME, we determined the percentage of MMS students who scored at or below the tenth percentile of first-time national examinees.

**Results:**

MCAT scores correlated robustly with USMLE 1 and 2, and USMLE 1 and 2 independently predicted NBME scores in all clerkships. USMLE 1 cutoffs corresponding to poor NBME performance ranged from 220 to 223. USMLE 1 scores were similar among MMS and visiting students. For most academic years and clerkships, NBME scores were similar for MMS students vs all first-time examinees.

**Conclusions:**

MCAT, USMLE 1 and 2, and subsequent clinical performance parameters were correlated with NBME scores across all core clerkships. Even more interestingly, faculty assessments correlated with NBME scores, affirming patient care as examination preparation. USMLE 1 scores identified students at risk of poor performance on NBME subject examinations, facilitating and supporting implementation of remediation before the clinical years. MMS students were representative of medical students across the nation.

## Background

Standardized examinations are ubiquitous throughout medical education and are designed to objectively measure performance. Unsurprisingly, performance on a given standardized test tends to predict performance on subsequent tests. The Medical College Admission Test (MCAT) is widely used to select students with a higher likelihood of success in medical school, and it is considered more valid than letters of recommendation and grade point averages (GPAs) [1, 2]; MCAT performance also predicts performance on the United States Medical Licensing Examination Step 1 (USMLE 1) [3]. USMLE 1, administered after the first 2 years of medical school, assesses understanding and the ability to apply important concepts in basic science to the practice of medicine. It covers traditional disciplines such as anatomy, behavioral sciences, biochemistry, biostatistics and epidemiology, microbiology, pathology, pharmacology, and physiology, as well as interdisciplinary areas such as genetics, aging, immunology, nutrition, and molecular and cell biology [4]. The National Board of Medical Examiners (NBME) comprehensive basic science subject examination also can help identify students at risk for failing USMLE 1 [5, 6]. MCAT and USMLE 1 scores are also predictive of NBME obstetrics and gynecology (OB/GYN) subject examination scores [7].

Further in medical education, several studies link poor test results with lower performance during the clinical years, and moderate-to-strong correlations were identified between NBME subject examination scores and USMLE performance [8, 9]. For example, students failing either the USMLE 1 or NBME OB/GYN subject examination were more likely to fail USMLE 2 [10]. USMLE 2 (Clinical Knowledge and Clinical Skills), taken during the fourth year of medical school, assesses application of medical knowledge, skills, and understanding of clinical science that are essential for providing patient care under supervision. It includes questions regarding the immune system, blood and lymphoreticular systems, behavioral health, nervous system and special senses, skin and subcutaneous tissue, musculoskeletal system, cardiovascular system, respiratory system, gastrointestinal system, renal and urinary systems, pregnancy, childbirth and the puerperium, female reproductive system and breast, male reproductive system, endocrine system, multisystem processes and disorders, biostatistics, and epidemiology/population health, as well as interpretation of the medical literature [11, 12]. Poor performance on the third-year NBME surgery subject examination was strongly correlated with the second-year pathology NBME subject examination scores [13, 14]. Lastly, examinees with low USMLE scores also had a higher risk of failing part 1 of the American Board of Orthopaedic Surgery Certifying Examination [15]. Poor standardized test scores before the clinical years may identify students who would benefit from proactive support to avoid poor performance and thereby increase their ability to successfully complete clerkships. In addition, student assessments do not need to be limited to standardized tests. For example, a faculty-developed pretest given on day 1 of an internal medicine clerkship reliably identified students at risk for poor performance [16].

Curriculum design also affects standardized test scores. Decreasing the duration of an OB/GYN medical student clerkship resulted in lower subject examination scores, especially for students enrolled in the first half of the academic year [17]. Similarly, shortening the psychiatry clerkship length at Mayo Medical School (MMS) negatively affected NBME scores [18]. Certain clerkship characteristics are associated with better student examination performance, the most important being caring for more patients per day [19]. Self-assessment practice examinations can accurately predict actual performance on standardized tests, although some variation in predictive accuracy occurs across different test administration conditions [5, 20].

Medical schools strive to produce inquisitive physicians with a basic foundation of knowledge, technical skills, reasoning ability, and empathy; this provides a sufficient framework for graduate medical education and practice and helps develop skills for self-directed lifelong learning. Factors that predict a student’s success in all these areas are difficult to identify [21]. Critics of standardized examinations point out that the tests primarily measure medical knowledge and may be poor predictors of clinical performance and professionalism [22, 23]. Not surprisingly, a survey of students reported that those who scored well on numerically scored standardized tests were more likely to favor their use, whereas those who scored poorly favored pass/fail grading [24, 25].

Although several studies have confirmed correlations between various standardized tests, less is known regarding the relationship between standardized test results and clinical performance. In fact, subjective assessment of surgical knowledge by faculty and residents correlated poorly with objective performance measures, bringing into question whether subjective appraisal of surgical knowledge should be included in the assessment process [26]. Nevertheless, others have reported that grades from an OB/GYN rotation correlated with USMLE scores [27] and NBME subject examination scores [7].

The merits of using either objective standardized testing or subjective clinical assessments can be debated, but both remain common components of medical student assessment. We asked the following questions: 1) How do test scores relate to medical student clinical performance? and 2) How do faculty assessments relate to overall student performance on core clerkships? Our study focused on standardized testing relationships throughout medical school as they relate to performance on all clinical clerkships. If longitudinal and cross-sectional relationships between standardized testing (both before and during medical school) and clinical performance can be confirmed, poor performers may be identified and supported before their clinical years.

## Methods

We included students who matriculated at MMS from 2000 through 2009. We selected 2009 as the last year of matriculation to allow access to complete information across all 4 years of medical school. The study was deemed exempt by the Mayo Clinic Institutional Review Board (protocol 13-003310), and data were de-identified before analysis.

The following information was obtained for each student: gender, age, underrepresented minority status, degree program, undergraduate GPA, MCAT subscores and total score, USMLE 1 and 2 scores, NBME subject examination scores (range, 0–100), and faculty assessment scores (FAS) (range, 1–5, with 5 representing the highest possible score). The subcategories of faculty assessment encompassed domains of clinical knowledge, including history taking, examination skills, and decision making. Further, professional behaviors such as communication, teamwork, and patient-centered approach were assessed. In addition, we obtained FAS and USMLE 1 scores of visiting medical students completing clerkships (in internal medicine, neurology, OB/GYN, pediatrics, surgery, family medicine, psychiatry, and emergency medicine) from August 2006 through August 2012. All data were electronically retrieved from databases maintained by the MMS.

For MMS students, correlations between measures were evaluated graphically and quantified using the Pearson correlation coefficient. Multivariable linear regression models were fit using backward variable elimination to identify sets of independent predictors of USMLE 1, USMLE 2, and each of the NBME subject examinations, respectively. Predictors with a *P* value < .05 were retained in each final model.

Lastly, MMS students were compared with other medical students in 2 ways. First, FAS and USMLE 1 scores were each compared between MMS matriculating students and visiting medical students who concurrently completed the same clerkships. Scores were compared using the 2-sample *t* test. Second, using national data on NBME first-time examinees, we stratified students by clerkship and determined the overall percentage of MMS matriculating students who scored at or below the 10th national percentile for that academic year on the NBME subject examination from 2003–2012 [28].

Analyses were performed using SAS version 9.2 (SAS Institute Inc). All calculated *P* values were 2-sided, and *P* values less than .05 were considered statistically significant.

## Results

We identified 435 students who matriculated in 2000 through 2009 and completed USMLE 1. Of the 435 students, 219 (50.3 %) were men; 65 (14.9 %) were underrepresented minorities. The majority (*n* = 356 [81.8 %]) were in the standard MD program, the rest were in joint-degree programs for MD-PhD (*n* = 53 [12.2 %]), MD-DDS (*n* = 19 [4.4 %]), or MD-PhD-MS (*n* = 7 [1.6 %]) degrees. The results are reported for all programs combined.

Table 1 summarizes the observed correlation of student characteristics, undergraduate GPA, and MCAT scores with USMLE 1 scores. The strongest predictor of USMLE 1 was the MCAT total score (*r* = 0.50; *R*^*2*^ = 25 %). In a multivariable analysis using backward variable elimination, the following variables were identified as independent predictors of USMLE 1 (*P* values < .05) with an overall R^2^ of 35 %: GPA, MCAT–biological science, MCAT–physical science, and gender. As previously described [29], USMLE 1 scores were slightly higher for men than women. Table 1 also summarizes the observed correlations with USMLE 2 clinical knowledge scores (available for 324 students). Overall, the strongest predictor of the USMLE 2 score was the USMLE 1 score (*r* = 0.77; *R*^*2*^ = 59 %). In a multivariable analysis using backward variable elimination, the following variables were identified as independent predictors of USMLE 2, with an overall *R*^*2*^ = 61.6 %: USMLE 1, gender, and MCAT–total.

**Table 1:** Correlation of test measures and student sharacteristics with USMLE 1 and 2

Test measure	Analysis of USMLE 1		Analysis of USMLE 2	
	r^a^	*P* value^b^	r^a^	*P* value^b^
GPA	0.39	<.001	0.35	<.001
MCAT				
Total	0.50	<.001	0.38	<.001
Biological science	0.49	<.001	0.37	<.001
Physical science	0.47	<.001	0.36	<.001
Verbal	0.21	<.001	0.19	<.001
Writing	0.16	<.001	0.11	.06
USMLE 1	…	…	0.77	<.001
Student Characteristic	USMLE 1, mean (SD)	*P* Value^c^	USMLE 2, mean (SD)	*P* Value^c^
Gender		<.001		.55
Male	236.3 (20.9)		236.1 (23.6)	
Female	228.6 (20.5)		237.6 (22.4)	
Underrepresented minority		<.001		<.001
No	235.0 (19.7)		239.1 (21.2)	
Yes	218.3 (23.3)		224.2 (28.5)	

*Abbreviations*: *GPA* Grade point average, *MCAT* Medical college admission test, *USMLE 1 and 2* United States medical licensing examination steps 1 and 2

^a^r denotes the Pearson correlation coefficient

^b^ *P* values indicate whether the correlation is significantly different from 0

^c^ *P* values were based on a 2-sample *t* test

Table 2 summarizes correlations of the test measures and student characteristics with NBME subject examinations, based on 407 students with at least 1 NBME subject examination score. The variables that independently correlated with each NBME score (based on a multivariable analysis) are shown. Both USMLE 1 and 2 independently correlated with NBME scores across clerkships.

**Table 2:** Correlations of GPA, MCAT, and USMLE scores with NBME subject examination scores for each clerkship

Test measure	Medicine		Neurology		OB/GYN		Pediatrics		Psychiatry		Surgery	
	r^a^	*P* value^b^	r^a^	*P* value^b^	r^a^	*P* value^b^	r^a^	*P* value^b^	r^a^	*P* value^b^	r^a^	*P* value^b^
GPA	0.33	<.001	0.31	<.001	0.29	<.001	0.38	<.001	0.32	<.001	0.35	<.001
MCAT												
Total	0.38	<.001	0.40	<.001	0.33	<.001	0.39	<.001	0.40	<.001	0.45	<.001
Biological science	0.36	<.001	0.38	<.001	0.33	<.001	0.34	<.001	0.35	<.001	0.43	<.001
Physical science	0.31	<.001	0.33	<.001	0.29	<.001	0.32	<.001	0.31	<.001	0.39	<.001
Verbal	0.23	<.001	0.26	<.001	0.17	<.001	0.27	<.001	0.30	<.001	0.26	<.001
Writing	0.10	.05	0.13	.01	0.09	.07	0.14	.006	0.21	<.001	0.12	.23
USMLE 1	0.69	<.001	0.69	<.001	0.65	<.001	0.68	<.001	0.62	<.001	0.67	<.001
USMLE 2	0.77	<.001	0.75	<.001	0.74	<.001	0.77	<.001	0.69	<.001	0.70	<.001
Student Characteristic	Mean (SD)	*P* Value^c^	Mean (SD)	*P* Value^c^	Mean (SD)	*P* Value^c^	Mean (SD)	*P* Value^c^	Mean (SD)	*P* Value^c^	Mean (SD)	*P* Value^c^
Gender		.77		.54		.08		.26		.02		.26
Male	78.4 (8.3)		75.2 (8.2)		74.5 (8.8)		76.4 (9.1)		77.2 (9.0)		75.6 (9.5)	
Female	78.2 (8.0)		75.7 (8.2)		76.0 (8.3)		77.4 (9.3)		79.2 (8.9)		74.6 (8.9)	
Underrepresented minority		<.001		<.001		<.001		<.001		<.001		<.001
No	79.1 (7.8)		76.2 (7.7)		76.0 (8.4)		77.9 (8.7)		79.0 (8.7)		75.9 (9.1)	
Yes	73.9 (8.6)		70.9 (9.1)		70.9 (8.2)		71.6 (10.1)		73.7 (9.7)		70.3 (8.4)	
Independent predictors from multivariable models	USMLE 2		USMLE 2		USMLE 2		USMLE 2		USMLE 2		USMLE 2	
	USMLE 1		USMLE 1		USMLE 1		USMLE 1		USMLE 1		USMLE 1	
	MCAT, Physical		MCAT, Physical		Gender		Gender		Gender		MCAT, biological science	
	MCAT, Total		MCAT, Total				MCAT, Verbal		MCAT, Physical			
									MCAT, Total			
	(*R* ^*2*^ = 60.6 %)		(*R* ^*2*^ = 59.0 %)		(*R* ^*2*^ = 58.4 %)		(*R* ^*2*^ = 63.0 %)		(*R* ^*2*^ = 53.4 %)		(*R* ^*2*^ = 52.6 %)	

*Abbreviations*: *GPA* Grade point average, *MCAT* Medical college admission test, *NBME* National board of medical examiners, *OB/GYN* Obstetrics and gynecology, *USMLE 1 and 2* United States medical licensing examination steps 1 and 2

^a^r denotes the Pearson correlation coefficient

^b^ *P* values indicate whether the correlation is significantly different from 0

^c^ *P* values were based on a 2-sample *t* test

Of the 435 students in the cohort, FAS were available for 222 students (available for 27/211 students [12.8 %] who matriculated in 2000–2004 and 195/224 students [87.1 %] who matriculated in 2005–2009). Table 3 summarizes correlations between FAS and the MCAT, USMLE, and NBME scores for each clerkship. FAS correlated well with USMLE 1 and 2 across almost all clerkships. NBME and FAS also correlated well across all clerkships. FAS was not correlated with MCAT scores.

**Table 3:** Correlation of Clerkship Faculty Assessment Score with MCAT, USMLE, and NBME Scores

Clerkship	MCAT, total		USMLE 1		USMLE 2 clinical knowledge		NBME	
	r^a^	*P* value^b^	r^a^	*P* value^b^	r^a^	*P* value^b^	r^a^	*P* value^b^
Medicine	0.08	.26	0.26	<.001	0.14	.06	0.30	<.001
Neurology	*−*0.05	.49	0.20	.007	0.20	.009	0.25	<.001
OB/GYN	*−*0.02	.75	0.15	.04	0.12	.11	0.22	.002
Pediatrics	*−*0.08	.30	0.13	.07	0.20	.009	0.18	.01
Psychiatry	0.03	.68	0.19	.01	0.22	.003	0.30	<.001
Surgery	*−*0.02	.77	0.18	.02	0.11	.15	0.16	.03
Family medicine	0.20	.009	0.37	<.001	0.26	.001	…^c^	
Emergency medicine	0.06	.37	0.20	.005	0.16	.03	…^c^	
Internal medicine subspecialties	0.12	.12	0.22	.003	0.15	.04	…^c^	

*Abbreviations*: *MCAT* Medical college admission test, *NBME* National board of medical examiners, *OB/GYN* Obstetrics and gynecology, *USMLE 1 and 2*, United States medical licensing examination steps 1 and 2

^a^ r denotes the Pearson correlation coefficient

^b^ *P* values indicate whether the correlation is significantly different from 0

^c^ NBME subject examination scores were not available for some clerkships

Given the high correlation between USMLE 1 and NBME subject examination scores, we sought to determine a USMLE 1 cutoff that was predictive of poor performance on the NBME examinations, defined as a score at or below the 10th percentile. For each clerkship, we selected the USMLE 1 threshold at which both sensitivity and specificity were simultaneously maximized, to minimize the false-negative and false-positive rates. As summarized in Table 4, depending on the NBME clerkship, a USMLE 1 score ranging from 220 to 223 was predictive of poor performance on NBME subject examinations, with 77 % to 83 % sensitivity and specificity.

**Table 4:** USMLE 1 cutoff scores predictive of NBME subject examination scores below the 10th percentile for each core clerkship

NBME subject examination		USMLE 1 cutoff score^b^	Sensitivity, %	Specificity, %
Clerkship	10th percentile^a^			
Internal medicine	68	≤223	77.1	77.9
Neurology	65	≤222	77.8	79.7
OB/GYN	65	≤222	81.3	80.4
Pediatrics	65	≤222	80.0	79.9
Psychiatry	67	≤223	78.7	77.9
Surgery	63	≤220	82.6	83.0

*Abbreviations*: *NBME* National board of medical examiners, *OB/GYN* Obstetrics and gynecology, *USMLE 1* United States medical licensing examination step 1

^a^Ten percent of 435 students scored at or below this threshold

^b^Cutoff scores were selected to maximize sensitivity and specificity

Clerkships were completed by 755 visiting medical students from August 2006 through August 2012. The mean USMLE 1 was identical for the 435 MMS students and 207 visiting students for whom data were available (*P* = .99). The number of faculty assessments performed varied for each clerkship; however, mean FAS differed between the 2 groups only for OB/GYN (mean difference, 0.17; *P* = .05), surgery (mean difference, 0.17; *P* < .001), and family medicine (mean difference, 0.56; *P* = .04).

To benchmark MMS student performance on NBME subject examinations against the national pool of first-time examinees, we determined the percentage of MMS students from each clerkship who scored at or below the 10th national percentile for that academic year on the NBME subject examination from 2003 through 2012. The overall percentages were 5.5 % (95 % CI, 3.0-8.0 %) for OB/GYN, 7.1 % (95 % CI, 4.2-10.0 %) for both surgery and neurology, 7.7 % (95 % CI, 4.8-10.7 %) for internal medicine, 9.4 % (95 % CI, 6.0-12.9 %) for pediatrics, and 11.7 % (95 % CI, 8.1-15.3 %) for psychiatry.

## Discussion

### Medical student performance during the clinical years is predictable across clerkships

We examined the feasibility of predicting performance across core clerkships by examining relationships among examination scores and clinical performance parameters before and during medical school. Clinical performance encompassed FAS and various other aspects of learning that were specific to each clerkship, including demonstrated skills, topic presentations, case summaries, and reflections. We included MCAT scores, undergraduate GPA, USMLE 1 and 2 scores, NBME subject examination scores, and FAS. We confirmed several previously reported relationships, including strong correlation of all subsections of the MCAT with USMLE 1. Further, all MCAT subsections except writing were highly correlated with USMLE 2 (Tables 1 and 2). The latter finding is less relevant because the future MCAT format will not include any writing [30]. Of primary interest to our anticipated outcomes, NBME subject examination scores across all clerkships, not reported previously, were highly correlated with USMLE 1 and 2 scores, whereas undergraduate GPA and MCAT had less robust correlations.

The next, less expected finding is educationally relevant and reflects the benefits of thoughtful, high-quality faculty feedback. When considering students from all core clerkships for whom NBME subject examination scores were available, FAS robustly correlated with NBME examination scores, more so than they did with MCAT or USMLE 1 and 2 scores, although for most clerkships, FAS and USMLE scores also were correlated (Table 3). At our institution, students frequently describe tension between patient care responsibilities during clerkships and the need to master material tested on the NBME subject examinations (“Do we study for the test or take care of patients?”). To the extent that faculty assessments are based on observations of students taking care of patients, we are now able to support our answer to this question with evidence—taking care of patients is excellent preparation for the test.

Although the MCAT total score had high predictive value for USMLE 1, USMLE 2, and NBME examination scores, it was not well correlated with core clerkship FAS. In fact, our data suggest the absence of a relationship between MCAT score and FAS (Table 3), even though MCAT and FAS correlated well with performance on the corresponding NBME examination. This finding may warrant further inquiry into which aspects of the MCAT predict success in clinical medicine.

We investigated the predictive value of standardized tests in identifying students who may need additional help or support during the clinical years (Table 4). We chose the 10th percentile as a cutoff because it is identical to the minimum passing NBME subject examination score in core clerkships at MMS. We outline, with maximal sensitivity and specificity, the USMLE 1 score below which we expect to see an NBME subject examination score in the lowest 10th percentile on each clerkship. The cutoff scores varied by clerkship, but the USMLE 1 may facilitate identification of at-risk students before clerkships begin, which potentially allows proactive enhanced support before and during the clerkship, rather than retroactive remediation after failure of the clerkship. The nature of such remediation is beyond the scope of this work, but determining parameters most relevant for identifying students potentially at risk is an educationally sound first step.

Given the small class size at MMS, we evaluated the generalizability of these data beyond comparison of standardized scores. Because FAS in this study are specific to our institution, comparison of MMS students with visiting students showed that USMLE 1 scores were identical, confirming a similar level of knowledge. FAS are inherently subjective, and they varied among MMS students in some clerkships (family medicine, OB/GYN, pediatrics, and surgery) but not in others (internal medicine, neurology, and emergency medicine). Given this variability among clerkships and the relatively small sample sizes for individual clerkships (but not for the overall comparison), we do not believe that these relatively small differences are educationally meaningful, particularly given the absence of an overall trend. We believe that the MMS cohort adequately represents US medical students. Therefore, whereas the calculation of a USMLE 1 cutoff predictive of lower clerkship performance is institution-specific, the method we used could be easily replicated at other medical schools using available data. These predictors also may provide meaningful guidance to the admissions process and curriculum design.

### Strengths

The key strengths of this study include the longitudinal and cross-sectional correlations between standardized scores and clinical performance parameters across all core clerkships. Unlike earlier studies that focused on single clinical clerkships [7, 15, 18, 19, 27, 31, 32], we analyzed scores and clinical assessments across all required clerkships. These findings may allow us to identify students with need of assistance in improving performance in any of the core clerkships. We also identified clinical acumen in terms of FAS as a vital contributor to overall clinical performance. This finding is both novel and educationally meaningful. Lastly, we generalized MMS data through comparison with clinical assessments of visiting medical students from across the United States and comparison with national NBME standards for the subject examinations pertaining to each clerkship.

### Limitations

Limitations include a retrospective design at a single institution over a long study period. Some variation in scores may have been introduced by a curriculum change in 2006, although we included similarly sized groups of students enrolled before and after the change. Further, not all scores were available for all students in all clerkships during the study interval.

## Conclusions

In summary, we described academic assessment of MMS students over a 10-year interval. We confirmed a number of well-established findings regarding the predictive value of MCAT, USMLE 1 and 2, and NBME examinations in individual clerkships while expanding to report trends across all core clerkships. We also showed that both standardized testing and FAS were highly predictive of NBME subject examination scores, whereas MCAT scores did not correlate well with FAS in the clinical years. We confirmed that USMLE 1 sensitively and specifically identified students at risk for poor performance in clerkships, before clerkships began. Our findings can facilitate identification of students at risk for poor clinical performance who would potentially benefit from proactive remediation. Because the MMS cohort was comparable in terms of the primary outcomes of interest (NBME and FAS) to other US medical students, we believe that our findings are generalizable and that the methodology can be replicated to determine institution-specific performance cutoffs.

### Consent to publish

No potentially identifying details are included in the text. Consent to publish is not applicable for this manuscript.

### Availability of data and materials

The dataset supporting the conclusions of this article is included within the article.

## Abbreviations

FAS: faculty assessment scores
GPA: grade point average
MCAT: Medical college admission test
MMS: Mayo medical school
NBME: National board of medical examiners
OB/GYN: obstetrics and gynecology
USMLE 1 and 2: United States medical licensing examination steps 1 and 2
